# Ice crystals growth driving assembly of porous nitrogen-doped graphene for catalyzing oxygen reduction probed by *in situ* fluorescence electrochemistry

**DOI:** 10.1038/srep06723

**Published:** 2014-10-22

**Authors:** Jiong Wang, Huai-Song Wang, Kang Wang, Feng-Bin Wang, Xing-Hua Xia

**Affiliations:** 1 State Key Laboratory of Analytical Chemistry for Life Science, School of Chemistry and Chemical Engineering, Nanjing University, Nanjing 210093, China

## Abstract

In recent years, doped carbonaceous materials as alternative catalysts for oxygen reduction reaction (ORR) have received considerable attention due to the low cost and high CO tolerance capability. Different theoretical studies have suggested that oxygen is reduced in a rapid sequence intermediated by diverse oxygen-containing reactive intermediates (ORI). However, due to the short lifetimes of the possible ORI, direct experimental evidence is very difficult to be obtained. Here, we report the synthesis of an ultralight and porous nitrogen-doped graphene (NG) by annealing graphite oxide (GO)-melamine scaffold shaped in ice template. The resultant NG exhibits excellent electrocatalytic activity toward *4e*-reduction of oxygen with the onset potential as low as −0.05 V *vs.* Ag/AgCl in alkaline media. Using this material as model study, sensitive *in situ* fluorescence spectroelectrochemistry is applied to demonstrate the presence the reactive ORI. The global ORR pathway is unraveled as stepwise electron transfer involving hydroxyl radical as the important intermediate via both inner- and outer-sphere process. This result would likely provide a new insight into the further understanding of ORR mechanism on those intrinsic carbonaceous materials.

The continuous growth of global energy crisis requires the development of renewable energy conversion systems. Among them, fuel cells have been attracting widespread attention due to the effective energy conversion capacity and elimination in pollution emission^1^,^2^. As the half-cell reaction, the sluggish oxygen reduction reaction (ORR) demands more amount of efficient catalysts to activate the stable O-O bond (498 kJ/mol^3^) comparing to the faster anodic hydrogen oxidation. Although Pt and Pt-based alloys exhibit high specific activity and low overpotential towards ORR (200 μA/cm^2^_Pt_ at 0.9 V, Pt/C catalyst^4^), the real application of this metal is currently hampered by high cost and low earth storage.

Efforts to develop alternative catalysts have been focused on the carbonaceous materials mainly for two reasons. First, such alternations satisfy the basic physicochemical requirements, conductivity and stability, for running a real fuel cell system. Second, the breakthrough in ORR activity has recently been achieved by the N doping strategy^5^,^6^,^7^,^8^. A typical example is performed on aligned N-doped carbon nanotubes, where the catalytic current density is up to 4.1 mA/cm^2^ (−0.22 V *vs.* Ag/AgCl, air-saturated 0.1 M KOH)^9^. Based on the commonly accepted inner-sphere mechanism, the decrease in overpotential indicates a preferred O_2_ adsorption on the catalyst. Essentially, in the molecular-orbital viewpoint, this phenomenon suggests that the Fermi energy of *sp*^*2*^-carbon is well tuned by N, which reaches a certain level to match the energetic π-anti bond orbital in O_2_. DFT study^9^ has demonstrated the variation of O_2_ adsorption from linear- to bridge-mode upon the carbon atom polarized by the neighbouring N atoms. On the other hand, the electrochemical simulation proposed by Stevenson *et al*^10^ suggests that the onset potential should depend on the reaction rates of the followed elemental steps. DFT study by Bao *et al*^11^ shows that N in graphene lattice totally changes the minimum energy barriers of ORR elemental steps, which leads to a new global pathway. In this process, it shows that the contained oxygen intermediates are diverse, such as O^ ˙^, HO^ ˙^ or O^−^_2_ are all possible to exist. However, a direct experimental capture to identify these reactive species is rather difficult to be realized due to their short lifetimes. Accordingly, the fundamental understanding of ORR mechanism yet has not been fully understood, which blocks the further progress in overpotential decrease on N-doped carbonaceous materials. In this work, we take advantage of the faster kinetics of radical reaction to compete with the transient ORR at catalyst surface, showing that *in situ* fluorescence spectroelectrochemistry is effective to demonstrate the contained oxygen-containing reactive intermediates (ORI) during catalyzing ORR by carbonaceous materials. This approach provides a new insight into the probe of mechanism with very high sensitivity.

The carbonaceous model in this work is graphene, the well-known *sp*^*2*^-carbon array. Our latest researches have verified the significance of graphene in catalyzing a variety of substrates after appropriate modifications^12^,^13^,^14^,^15^. Melamine is well accepted as a cheap source for N doping and its solid state makes the N-doping more easily to be controlled comparing to the other N source (*i.e.* NH_3_). Simply by the pyrolysis of the powder hybrid of graphite oxide (GO) and melamine, the mass production of N-doped graphene thus becomes realizable. For the activity improvement towards ORR, the onset potential is shifted to −0.1 V *vs.* Ag/AgCl with *3.4e*- to *3.6e*-transfer in alkaline medium. But this N doping strategy brings a serious structural degrade that the specific surface area of graphene drastically decrease to ~ 6 m^2^ g^−1^ 16. Thus, an obvious larger catalytic current is not achieved. To overcome this barrier, a rational designing of hierarchical structure becomes the current challenge. Using ice templates, a novel N-doped graphene (NG) porous network is found to be constructed. Water is certainly the most powerful and common dispersant for solid deposits. It should be bore in mind that freezing directly makes water as a removable scaffold upon transferring to ice. Porosity is thus made as the dispersed particles are separated within the ice scaffold. By controlling the nucleation, tuned morphologies were created to satisfy various applications in sensors^17^, catalysis^18^, ceramic engineering^19^ and bionics area^20^. Herein, for the GO-melamine solid hybrid, we show this technique is quite useful to improve the specific surface area of final catalyst by producing porosity, as the proposed mechanism is presented in Figure 1. Making templates starts with freezing the vessel bottom for generating an instaneous thermal gradient inside liquid. At cold interface, firstly appeared ice pillars shift the dispersed GO/melamine and locally condense the suspension. Due to the colligative properties, the freezing point of water in this area sharply drops off to form a super-cooled region. The ice tips are forced to direct warm region where the nucleation is more favoured at the regular freezing point. In this way, growing ice crystals are controlled by the thermal gradient for making a mechanically strong architecture^21^,^22^,^23^. Brownian movements and the free falling of GO/melamine in this process are unavoidable. Both behaviours disturb the particles distribution, especially near the pillar tips, which is a great issue to the stabilities in shaping monolith^24^. In this regard, the freezing velocity is accelerated as much as possible by using liquid nitrogen (boiling point, ~77 K) as the cold source. After sublimating ice templates, there leaves a continuous bulk. X-ray diffraction (XRD) test on the bulk shows that GO nanoplates are isolated by melamine in ice crystals growth (Figure S4a). Nitrogen doping is subsequently conducted by heat treatment while the bulk form is preserved. In this strategy, melamine may be viewed as a second solid template since the excess amount is simultaneously gasified by heating (Figure S4b).

## Results and Discussion

### The Effectiveness of Ice Template for NG Structural Improvements

**Figure 2:**
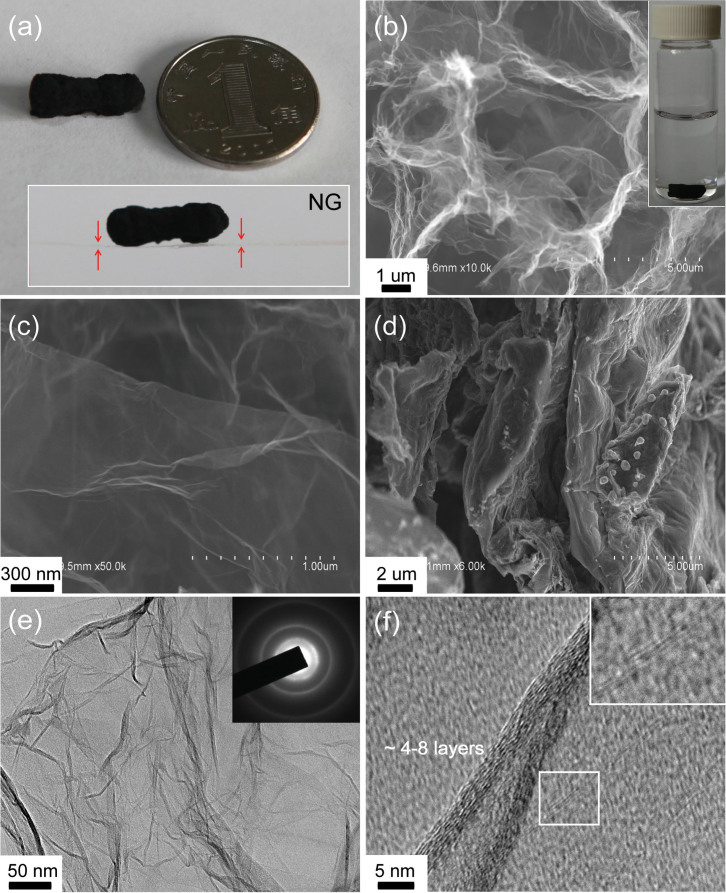
(a) Digital photos of NG monolith besides a Chinese coin of dime, and inset shows the bulk is placed on a lens wiping paper (~1 cm width, as denoted by the red arrows) suspending in air. (b, c) SEM images of NG, inset of (b) shows NG networks can readily sink in water. (d) Typical SEM image of NNG for comparison. (e, f) TEM images of NG consisted of ~4 to 8 atomic layers. Inset of (e) is the corresponding electron diffraction pattern.

Figure 2a shows the digital photograph of the as-synthesized NG. Resulting from the successive removal of the occupied ice and melamine, the bulk exhibits the ultralight property. Placing it on a suspended lens wiping paper does not generate any visual bending deformation (Figure 2a inset). Under the scanning electron microscopy (SEM, Figure 2b, c), the detailed morphology of NG is depicted, showing a network interconnected with continuous macropores. Also, the transparent nano walls containing abundant wrinkle characters in NG are distinctly identified. For comparison, the controlled sample of N-doped graphene (NNG, which is synthesized without ice template but simply pyrolyzing the mixture of solid GO and melamine) presents the thick deposition, seriously stacking with layer-by-layer (Figure 2d). Transmission electron microscopy (TEM, Figure 2e, f) imaging confirms the graphene-like details and amplified resolution on side edges indicates that the template synthesized NG is constituted with ~4–8 atomic layers. Corresponding electron diffraction test presents ring patterns, suggesting that N dopants bring more structural irregularity into the hexagonal crystal lattice of graphene. The presence of such defects may affect electrical conductivity of NG, but is somewhat expected since ideally crystallized graphene is known chemically inert^25^.

**Figure 3:**
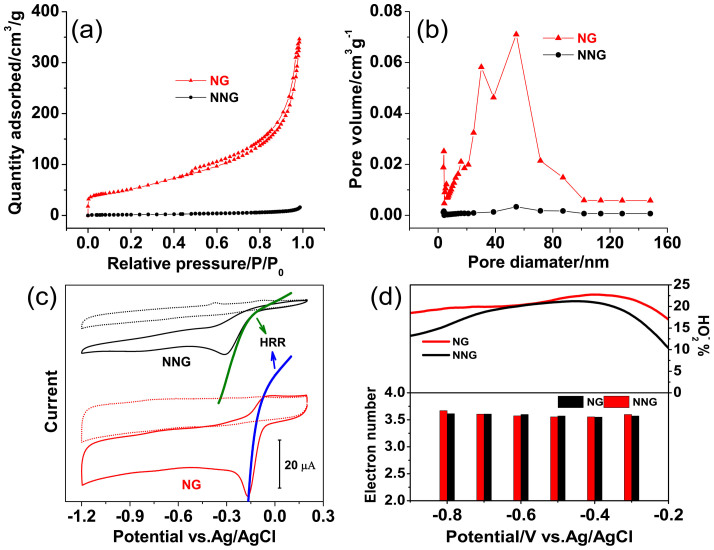
(a) Nitrogen adsorption/desorption isotherms of NG and NNG are tested at ~77 K. (b) The corresponding pore size distributions are analysed by Barrett-Joyner-Halenda (BJH) method. (c) Cyclic voltammograms of NG and NNG modified GC electrode (~loading 42.5 μg cm^−2^) in N_2_-purged (dotted) and O_2_-saturated (solid) 0.1 M KOH. The olive and blue curves stand for the corresponding HRR process on NNG and NG in the presence of 5 mM H_2_O_2_ tested in N_2_-purged 0.1 M KOH. (d) The corresponding peroxide yield and electron transfer number over the testing range, obtained from RRDE polarization curves.

Nitrogen adsorption/desorption isotherms of NG and NNG exhibit the type IV features (Figure 3a). Brunauer-Emmett-Teller (BET) analysis shows that the specific surface area of NG has increased up to 181.3 m^2^ g^−1^ comparing with 5.8 m^2^ g^−1^ of NNG. Furthermore, the inside pore channels play another key role in favouring molecule transport to heterogeneous catalysis. Barrett-Joyner-Halenda (BJH) calculation indicates large mesopores and macropores constitutes the void space of NG (20 to 80 nm, which is 49% of the total value estimated from the desorption branch). This superior size distribution is regard as making NG bulk readily sink in water despite of its hydrophobic surface (Figure 2b inset). The cumulative volume of pores (not including micropores, <2 nm, which is great hindrance for diffusion process as demonstrated in those zeolite frameworks^26^) constituted the inner void space of NG is 0.513 cm^3^ g^−1^. As the contrast, this value is only of 0.024 cm^3^ g^−1^ for NNG (Figure 3b).

The influence of the obvious structural variation from NNG to NG on catalysing ORR is tested by different electrochemical methods. In 0.1 M KOH, both materials exhibit substantial ORR process as compared to the featureless backgrounds in electrolyte saturated with N_2_. Remarkably, with loaded same mass, NG presents the much larger reduction peak and capacitive current (Figure 3c). Besides, a positive shift of onset potential from −0.11 V to −0.05 V is also observed. The study on platinum suggests that ORR both involves inner- and outer-sphere mechanism. In alkaline solution, the chemisorption of hydroxyl species on catalyst surface increases the probability of outer-sphere mechanism by forming HO^−^_2_ intermediate first^27^. In this process, O_2_ is solvated, not directly connecting with catalytic surface. Thereby, ORR activity would depend on the hydrogen peroxide reduction reaction (HRR). It is discovered in this study that the onset potential of ORR coincides with that of HRR. Especially on NG surface, HRR is somewhat more active than ORR, suggesting the outer-sphere mechanism is involved in catalysing ORR.

At the same diffusion rate, ORR polarization curves tested on the rotating disk electrode (RDE) confirm the less overpotential on NG, accompanied with the decrease in Tafel slope from 77 to 54 mV dec^−1^. (Figure S5) Thus, the higher exchange current of NG can be expected, which indicates the faster electron transfer rate in the early stage of ORR on NG. To verify the catalytic pathway, peroxide (HO^−^_2_) yield formed from *2e*-transfer pathway is measured by rotating ring-disk electrode (RRDE) experiments. Polarizing from −0.3 V to −0.9 V, HO^−^_2_ yields of both catalysts maintain low proportions, 22.5–18.6% for NG and 21.2–13.3% for NNG, corresponding to *3.58e*- and *3.59e*-transfer, respectively (Figure 3d). The decrease of HO^−^_2_ yields at larger overpotential can be primarily understood from HRR, where more HO^−^_2_ intermediate generated on disk would be further reduced at faster kinetics. And these results demonstrate that each reduced O_2_ contributes the same electric charges to ORR currents. Therefore, the increased current observed on NG should be resulted from the faster reaction kinetics and more electrochemically active sites inside the superior inner architecture. For comparison, graphene and Pt/C catalysts are tested under the same conditions. As expected, N dopants in graphene significantly improve both ORR activity (~100 mV decrease in overpotential) and selectivity of products (*0.9e*-enhancement). These performances are better than those of the most intrinsic N-doped carbonaceous materials reported in recent years (Table S1), closely approaching to the commercial Pt/C catalyst. Moreover, NG reflects the better durability with ~94% of current reserved after 4 h continuous running, whereas Pt/C exhibits ~80% current retention (Figure S6).

### The stepwise electron transfer of ORR demonstrated by *in situ* Fluorescence Spectroelectrochemistry

**Figure 4:**
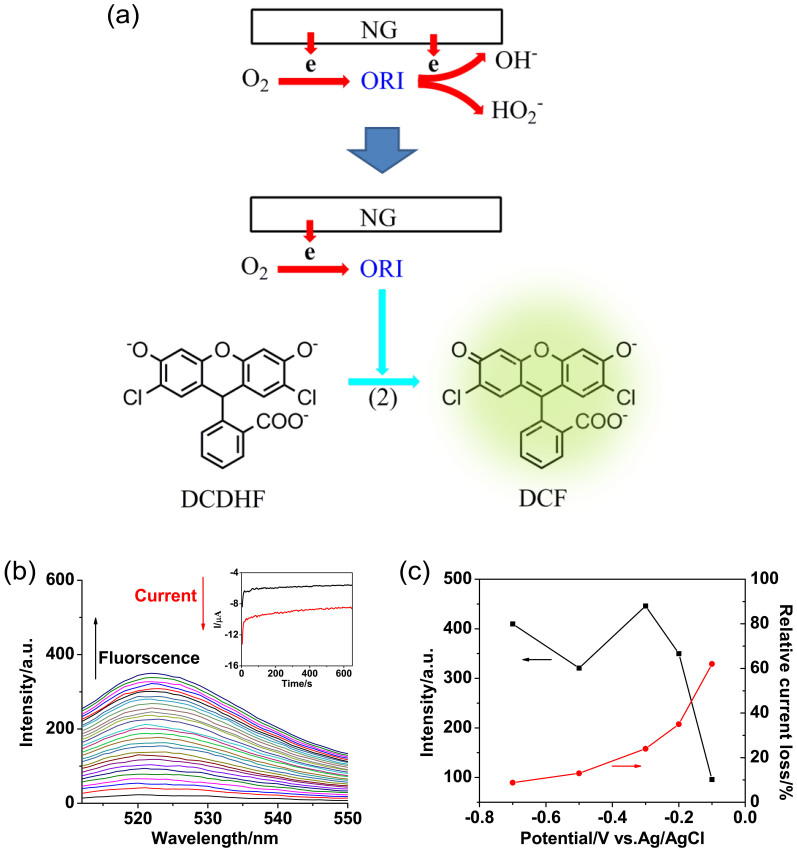
(a) Schematic illustration shows oxygen is reduced in rapid sequence on NG modified intermediated by ORI. Introducing a faster radical reaction allows capturing ORI during their lifetimes. Using a fluorescent probe, DCDHF, the capturing behaviour is reflected by the fluorescent signals. (b) Spectroelectrochemistry at −0.2 V of 2.5 μM DCDHF, in O_2_ saturated 0.1 M KOH. The electrolysis is lasted for ~650 s. (c) The summaries of fluorescent emission intensities and relative current loss at different overpotential.

Except that the HO^−^_2_ is considered as an intermediate in the outer-sphere mechanism, the detailed elemental steps in the global ORR pathway is further investigated by identifying more active ORI. Considering the high chemical reactivity of the possible intermediates contained in global pathway, the time taken for probing has to be shorter than their lifetimes. Thus, when oxygen is being reduced on NG modified electrode, a specific radical reaction is introduced to break the serial electron transfer, stabilizing the ORI in a new molecular form. The whole process is monitored by *in situ* fluorescence spectroelectrochemistry (Figure 4a).

The trapping agent of 2′,7′-dichlorodihydrofluorescin (DCDHF) is added into the electrolyte at potentiostatic potential of −0.2 V. Continuous growth of fluorescent emission at 522 nm is observed along with the electrolysis (Figure 4b). This is assigned to the entrapment of ORI by DCDHF, generating strong fluorescent dichlorofluorescin (DCF) (Figure S1, S2). In the corresponding i-t curve, the reduction current remains at −5.6 μA (~35% loss) in contrast to −8.7 μA without adding DCDHF, suggesting the serial ORR is partially blocked when the captured intermediates are supposed to further obtain electrons, forming OH^−^ or HO^−^_2_. In the presence of DCDHF, the trapping reaction competes with the global ORR, offering another pathway to consume ORI. Assuming the radical reaction rate is a constant, the capturing efficiency will be decreased with the increase of driving forces (*i.e.*, increasing the overpotential) for the electrochemical reduction of ORI. In our measurements, it is found that the relative current loss decreases with the increase of overpotential due to the increased reduction reactivity of intermediates, namely, it reaches maximum (62% loss) at −0.1 V, and decreases to 24% (−0.3 V), 13% (−0.5 V) and 8.8% (−0.7 V), demonstrating that less ORI are captured. On the other hand, the total current for ORR increases at higher overpotential, indicating the sum amount of the generated ORI has been increased in the radical reaction. For these two opposite effects, a peak shape curve of fluorescent emission versus overpotential appears with the strongest emission intensity at −0.3 V. Strangely, the fluorescent emission increases again at −0.7 V, possibly resulting from the involvement of second reduction process from HO^−^_2_ to OH^−^, and even the onset of water reduction (Figure 4c).

**Figure 5:**
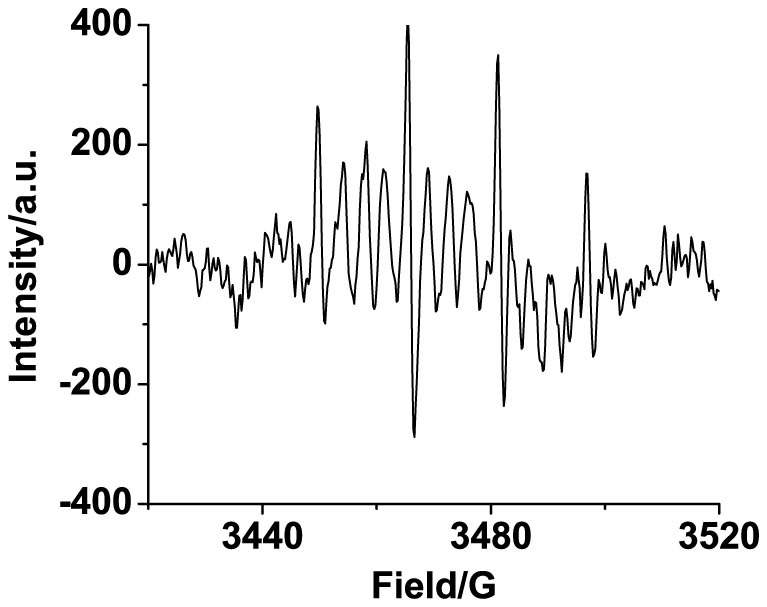
EPR spectrum of the solution after electrolysis of the NG modified electrode in O_2_-saturated 0.1 M KOH containing 170 mM DMPO for 1000 s shows the presence of HO^ ˙^ radicals.

The natures of ORI are probed by the 5,5-Dimethyl-1-pyrroline N-oxide (DMPO) signal molecule using electron paramagnetic response (EPR) spectrum. It shows that a distinct quartet at 3450, 3466, 3482 and 3497 G is appeared with the intensity ratio of 1:2:2:1, demonstrating that the intermediates are HO^ ˙^ radicals (Figure 5). The divided resonance peaks between quartet is assumed to be resulted from the K nucleus spin (I = 3/2) in KOH (Figure S9).

### Considering about ORR mechanism on NG

**Figure 6:**
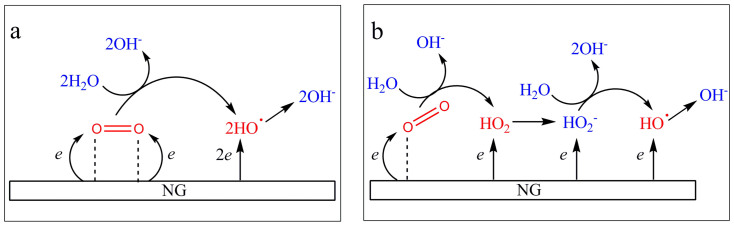
Schematic illustration of the proposed mechanisms of the parallel reaction pathways for ORR on NG catalyst. Mechanism (a) is considered as the principal reaction, and (b) as the side reaction.

Based on the results from *in situ* fluorescence spectroelectrochemistry and electrochemical measurements, it is believed that at least two parallel pathways are coexisted in ORR as two species of OH^−^ (~80%) and HO^−^_2_ (~20%) are finally formed on NG (Figure 6). Previous works using inner-sphere mechanism have suggested that O_2_ adsorption on inert catalysts prefers linear mode. In this case, the weakening of O-O bond is relatively difficult for there is only one O atom being connected for electron transfer^9^,^28^. An effective pathway calls as bridge-activated O_2_ when both O atoms have the equal chance to receive electrons from the electrode. Based on this perception and our experimental results, we propose the detailed ORR steps occurring on NG. One pathway for ORR follows the reactions 1 and 2 (E^0^ = 0.401 V *vs.* SHE). Since ORI have been identified as HO^ ˙^ radicals, the corresponding elemental step is proposed as reaction 3 (E^0^ = −0.024 V *vs.* SHE). In this step, O_2_ adsorption on NG adopts the bridge-activated mode, thus, both O atoms simultaneously obtain the electrons/protons, being analogues to the reaction at Pt catalyst. HO^ ˙^ can accept one additional electron from the electrode to form OH^−^ (reaction 4, E^0^ = 1.90 V *vs.* SHE), accomplishing the perfect *4e*-transfer^29^. It can also be consumed by chemical reaction, namely, trapped by DCDHF or annihilated by surrounding species (*i.e.*, water), resulting in imperfect *4e*-transfer. Thus, the electron transfer number (n) in this pathway varies, which is in good agreement with the results in Figure 3d. 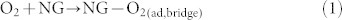

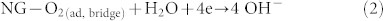


Elemental steps in B: 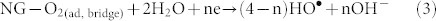

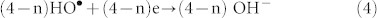


Another parallel pathway involves the formation of ~20% HO^−^_2_, as described in the chain reactions (5 and 6, E^0^ = −0.076 V *vs.* SHE) starting with the linear-activated O_2_ on NG. In this case, since only one O atom occurs electron transfer, O^−^_2_ is considered as the initial product (8, E^0^ = −0.624 V *vs.* SHE^30^), a strong Brønsted base. The initial product will be rapidly protonated by H_2_O (reaction 9) forming HO_2_. This species is in turn electrochemically reduced to HO^−^_2_ which is obviously more stable in alkaline medium (reaction 10, E^0^ = 1.5 V *vs.* SHE^31^). In this pathway, the global reaction occurs via a *2e*-transfer process. At larger overpotential, HO^−^_2_ reduction might increase the fluorescent emission as observed from *in situ* fluorescence spectroelectrochemistry. We consider reaction 7 (E^0^ = 0.88 V *vs.* SHE) may occur by receiving two electrons from the electrode step by step, as presented in 11 and 12. At the same time, this result also reflects the possible outer-sphere mechanism, where initiating ORR proceeds with the solvated O_2_ rather than linear-activating O_2_. The resultant product or intermediate whereas remains as HO^−^_2_ specie. Despite the details in forming HO^−^_2_ is not clear in this process, the followed elemental steps in reaction E are still applicable for this mechanism. 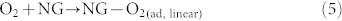

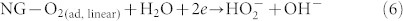

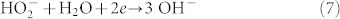
Elemental steps in D: 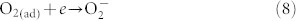

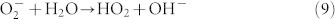

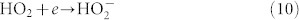
Elemental steps in E
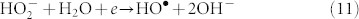

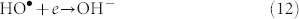


### Correlating catalytic activity with N species

**Figure 7:**
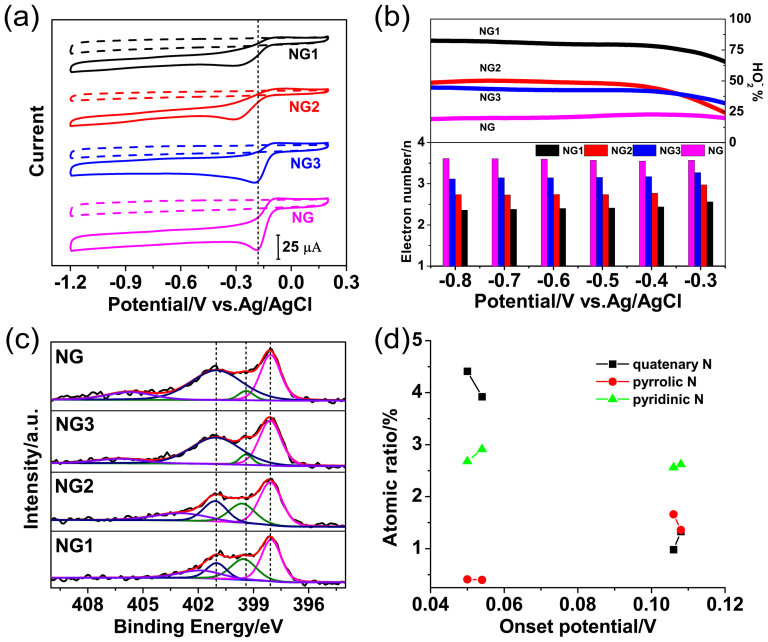
(a) Cyclic voltammograms of NG1, NG2, NG3, NG modified GC electrode in a N_2_-purged (dashed), O_2_-saturated (solid) 0.1 M KOH and (b) corresponding peroxide yields and electron transfer number per oxygen calculated by RRDE polarization curves. (c) High resolution N1s XPS spectra of NG1, NG2, NG3, NG. (d) The correlation of onset potential with different type of N species.

In the synthesis of NG, the mass ratio of melamine to GO in the initial hybrid is discovered as a major factor affecting the catalytic performance of final products. From the XRD patterns, the crystallinity of melamine is known considerably disrupted after blending with GO in aqueous solution except the case with far excess melamine added (Figure S4a). This is caused by the assemble behaviour between GO and protonated melamine through electrostatic interaction, *i.e.*, the formation of ionic links. The structural difference between ionic and non-ionic linked melamine may affect the configuration of the doped N in graphene, thus, three other catalysts, NG3, NG2 and NG1, are synthesized by gradually decreasing the mass ratios of melamine to GO from 17.6:1, 5:1 to 2.5:1. Electrochemical measurements show that less melamine leads to inert ORR activity and selectivity. For instance, ORR overpotential on NG1 is down shifted to −0.11 V and HO^−^_2_ yield is at ~80% over the polarization curve, corresponding to only *2.5e*-transfer (Figure 7a, b). (The activity degrade is also characterized by Koutecky–Levich plots in Figure S12).

XPS survey reveals the presence of C, N and O elements in above four materials (Figure S13). The proportion of melamine marginally affects N doping degree. Increasing melamine mass ratio by 13.3 times, N percentage in graphene only scales up from 6.22% to 8.54% while O content concomitantly decreases from 6.42% to 3.11%, suggesting N incorporation is realized by replacing some active O atoms (Table S2). Gaussian-Lorentzian analysis of the high-resolution N 1s XPS spectra show that N dopants feature different chemical conditions in graphene, being dominated with quaternary (N1, 401.06 ± 0.02 eV), pyrrolic (N2, 399.5 ± 0.2 eV), pyridinic (N3, 398.01 ± 0.02 eV)-like N and trace quantity of NO_x_ species (Figure 7c). Since onset potential directly stands for the decrease of activation energy (E_a_) for ORR catalysis, it is typically correlated with the atomic content of different N species. As presented in Figure 7d, with increasing onset potential, the conversion between N1 (4.41 to 0.98%) and N2 (0.41 to 1.66%) is observed, while N3 species remains almost constant. This result is also reflected by the accompanied structural variation. Since N2 is more hydrophilic than N1, surfaces of NG (126.1°) and NG3 (122.5°) exhibit more hydrophobicity than NG1 (105.2°) and NG2 (109.4°) (Figure S14). In the corresponding XRD patterns, *d*-spacing increases from 3.2 Å of pristine graphene to 3.3 Å of nitrogen doped samples as indicated by the down shift of 002 reflections, thus more accessible contacting sites are supplied. The XRD peak of NG is mostly distorted, indicating the doped N atoms mostly enter into the graphitic array rather than at edges or vacancies (Figure S15). Thereby, it is speculated that N1 species are more favourable to ORR, which coincides with the conclusions in many previous works^5^,^9^,^16^.

Further characterized by Raman spectra, two intense bands as the symbol of graphene structure are presented (Figure S16). D-band at ~1352 cm^−1^ is activated by the structural defects, indicating the breath mode of benzene^32^. In the presence of N, a uniform upshift of ~8 cm^−1^ on graphene is observed, reasonably reflecting the newly formed defects by foreign atoms^33^. The G-band at ~1589 cm^−1^ is attributed to the tangential vibrational mode of adjacent carbon atoms, revealing the information about internuclear distance. While N dopants only up shifts the G-band of NG1 and NG2 by 10 cm^−1^. This can be explained by the C-C strain or tension in carbon skeleton is affected by different C-N bonds, as can be seen from XPS analysis, suggesting that the modes of electron polarization between the doped N and neighbouring C atoms depend on their individual chemical natures. According to the previous study, N in some cases is not viewed as *n*-dopant for graphene^34^. Thus, the conversion from N1 to N2 would bring the different effect on the Fermi energy of graphene. Taking advantages of the molecular orbital theory^35^,^36^, such variation surely affects the catalytic activity, resulting from synergistic effects of different N species. From the experimental results in this study, with more N1 species being incorporated, the energy level of graphene can be optimum with matched with π-anti bond in O_2_ (or the energetic orbital of H_2_O_2_ when ORR is intermediated by this species proceeded with outer-sphere mechanism) for decreasing E_a_^37^. In addition, since HO^ ˙^ is found on NG that intermediates the ORR, it is suggested that the reasonable N distribution also tune the energy state of this species. This is speculated not only decrease to energy barrier of ORR, but also can increase the tendency for producing HO^−^ product with 4e-transfer. Finally, the improved catalytic performance of NG should be governed by the optimization of different materials properties. Except for the structure and doped N species characterizations, the electrical conductivity variation of NG is also measured because doping nitrogen in graphene is considered to increase the concentration of free charges carriers. This should bring a higher conductivity. Although it is commonly accepted that N doping decreases the crystallinity and in turn increases resistivity. Counter balance of these two effects, the conductivity of the least crystallized NG is still higher than that of the pristine graphene, showing that N dopants influence much more on the chargers carriers than the crystallinity (Figure S17).

## Conclusion

In this work, we have successfully improved the inner structure of N-doped graphene deposition by constructing a new three dimensional networks using ice crystals as templates. The specific surface area of the resultant NG has increased by 30-fold when the void volume is constituted by large mesopores and macropores (20–80 nm). Such structural variation facilitates the molecule transport, bringing ORR larger current accompanied with a decrease of overpotential of 60 mV. To demonstrate the possible oxygen-containing reactive intermediates (ORI) contained in the global ORR pathway, *in situ* fluorescence spectroelectrochemical method is developed. Both strong fluorescent signals and the loss of catalytic current suggest ORI in sequential electron transfer can be captured by a specific radical reaction. EPR measurement identifies the captured ORI as HO^ ˙^ radicals. Therefore, two parallel reaction pathways via the inner- and outer-sphere process on NG are proposed. Further, another three controlled NG samples are characterized in the same conditions to build a brief relationship between ORR activity and N functions. Results shows N species in graphene mainly include three forms and the conversion from pyrrolic- to quaternary-N enhances the catalytic performance. Due to the doping effect of N varies with their chemical natures, a rational content distribution of quaternary, pyrrolic and pyridinic-N leads a matched energy in graphene for O_2_ adsorption. These results would likely provide a new methodology for studying ORR on the intrinsic N-doped carbonaceous materials.

## Author Contributions

X.X. initiated the project and conceived the experiments. F.W. and K.W. helped to analysis the experiments data. H.W. designed and supplied the trapping agents for oxygen intermediates. J.W. performed all the experiments and wrote the manuscript together with X.X., all authors reviewed the manuscript.

## Supplementary Material

Supplementary Information Supplementary Info

## Figures and Tables

**Figure 1:**
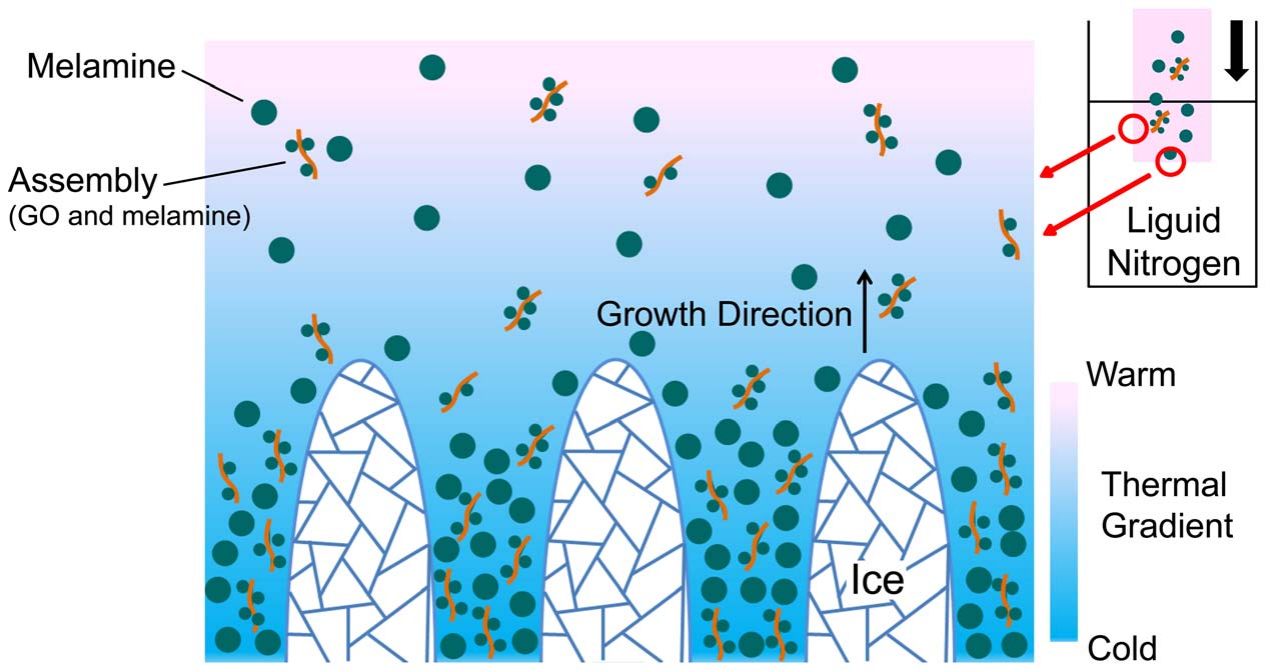
Schematic illustration of the proposed mechanism of ice crystals growth. As the added melamine is excessive in water (0.33 g/100 mL at room temperature), the acidic GO is assumed covered with a layer of protonated melamine, forming assemblies. To assess the solvability of the dispersed melamine, their grain sizes measured to be 6.885 μm (surface weighted mean), as shown in Figure S3a.
