# Supplementary material for: Ice crystals growth driving assembly of porous nitrogen-doped graphene for catalyzing oxygen reduction probed by in situ fluorescence electrochemistry
Source: Sci Rep. 2014 Oct 22;4:6723. doi: 10.1038/srep06723 (PMC4205891; doi:10.1038/srep06723)
Supplement: Supplementary Information — Supplementary Info [file srep06723-s1.pdf]

SUPPLEMENTARY INFORMATION

Ice crystals growth driving assembly of porous nitrogen-doped graphene for catalyzing oxygen reduction probed by *in situ* fluorescence electrochemistry

*Jiong Wang, Huai-Song Wang, Kang Wang, Feng-Bin Wang and Xing-Hua Xia**

State Key Laboratory of Analytical Chemistry for Life Science, School of Chemistry and Chemical Engineering, Nanjing University, Nanjing 210093, China

Reagents. Graphite powders (99.9995%, 100 mesh) were purchased from Alfa Aesar Company (UK). Melamine was purchased from Sinopharm Chemical Reagent Co. Ltd. (Shanghai, China). 2',7'-Dichlorodihydrofluorescin diacetate (DCDHF-DA) and 5,5-Dimethyl-1-pyrroline N-oxide (DMPO) were purchased from Sigma Company (USA). Other reagents were used as received (analytical-reagent grade). The aqueous solutions were prepared using Millipore water with a purity of 18.2 MΩ·cm.

Synthesis of 3D nitrogen-doped graphene (NG) network. The GO precursor was prepared using the method previously reported[1]. To synthesis NG, 5 mL aqueous dispersion of melamine (10 wt %) was mixed with 15 mL GO solution (0.1 wt %) by sonication to avoid air bubbles. The resultant suspension was frozen by dipping into the liquid nitrogen. Afterwards, the formed ice crystals were removed by sublimation below 0 ^{o}C. To achieve NG monolith, heat treatment of the dried product was carried out in a tube furnace under Ar atmosphere at 800 ºC for 1 h. The mass ratio of melamine to GO was varied to achieve different N doping percentage, with the products

labeled as NG1 (2.5:1), NG2 (5:1), NG3 (17.6:1). The synthesis of graphene was performed using the same procedure under the same conditions as preparing NG but without adding melamine. According to the published work[2], another controlled nitrogen-doped graphene was prepared to assess the influence of ice templating process on the properties of the product. Briefly, melamine and GO powders (mass ratio of 33.3:1, the same ratio as for NG) were mechanically mixed using an agate mortar. Nitrogen incorporation was performed by the same annealing design as above. The as-prepared product is label as NNG.

Apparatus. Atomic force microscope (AFM) images were acquired using an Agilent 5500 AFM/SPM system with Pico-scan v5.3.3 software. The samples were freshly prepared and drying on a mica plate. Imaging was performed in tapping mode under ambient conditions. Particle size distribution was tested on a Malvern Mastersizer 2000 particle size analyzer (Britian). The morphologies of each sample were characterized by transmission electron microscopy (TEM, JEM-200CX, Japan) by drying a droplet of sample suspension on Cu-grids. Scanning electron microscope (SEM) experiments were performed on S-4800 (Japan). The specific surface area and pore size distribution of the samples were calculated by Brunauer Emmett Teller (BET) analyses of nitrogen adsorption and desorption isotherm (ASAP2020, Micromeritics, USA). The oxygen-containing reactive intermediates were characterized by an electron paramagnetic resonance (EPR) spectrometer (EMX-10/12, Bruker). X-ray diffraction patterns and Raman spectra were carried out on a X'TRA (Switzerland) and a FT-Raman Spectrometer (Bruker), respectively. XPS spectra were obtained on a PHI 5000 VersaProbe (Japan). Curve fitting of the high resolution N1s spectra was performed using a ~20% Gaussian-Lorentzian peak shape. The

hydrophobic property was characterized on an optical contact angle measuring device (OCA30, Dataphysics Instruments Gmb).

Cyclic voltammetry (CV). The as-prepared product was dispersed in ethanol by at least 2 h of sonication to form a homogenous suspension with the concentration of 1.5 mg mL^{-1}. Then, 2 μL suspension was casted on a pretreated GC electrode (3 mm in diameter, ~ loading 42.5 μg cm^{-2}). A beaker was shield over the electrode so that the ethanol could evaporate slowly to form a uniform film. The measurements were carried out on a CHI 660D electrochemical workstation (CH Instrument Inc., USA) with Ag/AgCl electrode as the reference and a platinum wire as the counter electrode at a scan rate of 0.01 V s^{-1}. The electrolyte was 0.1 M KOH, saturated oxygen via bubbling O_2 gas at the beginning of each experiment. O_2 flow was maintained over the electrolyte during the measurements.

Rotation disk electrode (RDE) measurements. 4 μL suspension was casted on a pretreated GC electrode (4 mm in diameter, ~ loading 48 μg cm^{-2}) which is connected with a HP-1A system (purchased from Jiangfen Electrochemical Instrument Co. Ltd., China). The rest measurement system was the same as in CV measurements. The kinetic parameters were analyzed by the Koutecky–Levich equations[3]:

$$1/J_L + 1/J_K = 1/J \qquad \text{(S1)}$$

$$J_L = B\omega^{0.5},\ B = 0.2nFC_0D_0^{2/3}\upsilon^{-1/6} \qquad \text{(S2)}$$

where the J_L and J_K stand for the kinetic and diffusion limiting current densities, respectively. The measured current density is J. ω is the set rotation speed, n is the overall electron transfer number

for ORR, F stands for the Faraday constant (F=96485 C mol^{-1}), C_0 and D_0 stand for the concentration and diffusion coefficient of O_2 and υ the kinematic viscosity of the electrolyte.

Rotation ring disk electrode (RRDE) measurements. A four-electrode system was tested on a CHI 900D (CH Instrument Inc., USA). The working electrodes were composed by a glassy carbon as a disk (4 mm in diameter) and Pt as a ring (1 mm in width). Suspension was carefully casted on the disk without touching the ring. The potential on disk was varied with a scan rate of 0.01 V s^{-1} and the ring potential was set to be a constant value of 0.4 V *vs.*Ag/AgCl reference. The collection efficiency (N) was calculated by the ratio of ring current to disk current with 1 mM potassium ferricyanide as the probe in 0.1 M KCl medium. The rest measurement systems were the same as in RDE measurements. The peroxide yields and electron transfer number (n) were calculated by the following equations[4]:

$$HO_2^- \% = 200 \times (Ir/N)/(Id + Ir/N) \qquad (S3)$$

$$n = 4 \times Id/(Id + Ir/N) \qquad (S4)$$

where *Ir* and *Id* are the ring and disk currents, respectively.

***In situ* fluorescence Spectroelectrochemistry.** A three-electrode system combined with a fluorescence spectrophotometer (RF-5301PC, Shimadzu) was established to realize the real-time tracing radical oxygen species (ROS) during ORR. 2’,7’-Dichlorodihydrofluorescin (DCDHF) was chosen as the trapping agent. After cleaving the acetate groups in 2’,7’-Dichlorodihydrofluorescin diacetate (DCDHF-DA) in alkaline solution, this agent would readily react with ROS and convert to strongly fluorescent dichlorofluorescin (DCF) compound.

The formation of DCF was monitored by detecting the emission at 522 nm with excitation at 502 nm.

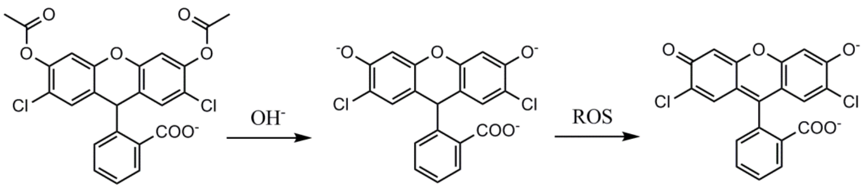

Figure S1. Schematic illustration of the mechanism of producing dichlorofluorescin (DCF) probe (DCDHF-DA) from 2',7'-Dichlorodihydrofluorescin diacetate (DCDHF-DA).

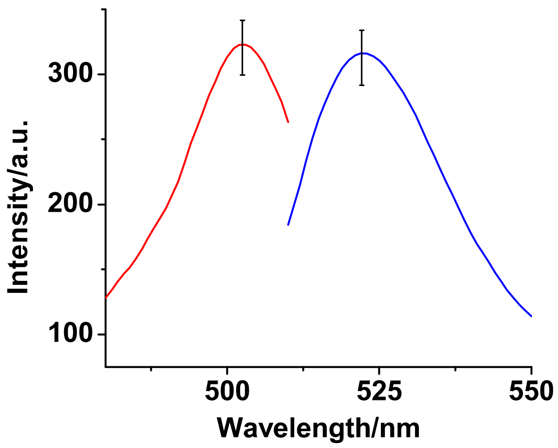

Figure S2. Fluorescence emission (blue curve, peak at 522 nm)/excitation (red curve, peak at 502 nm) spectra of dichlorofluorescin (DCF) obtained in 0.1 M KOH solution.

Characterization results

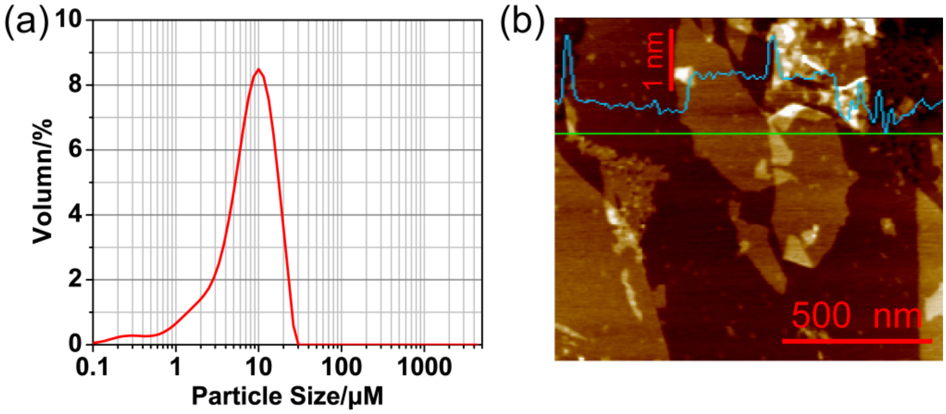

Figure S3. To verify the solvability, the grain size of melamine is measured as 6.885 μm (surface weighted mean), corresponding to the specific surface area of 0.871 $m^2 g^{-1}$. (b) AFM image shows that GO has a thickness of 0.5 nm with 500 nm width.

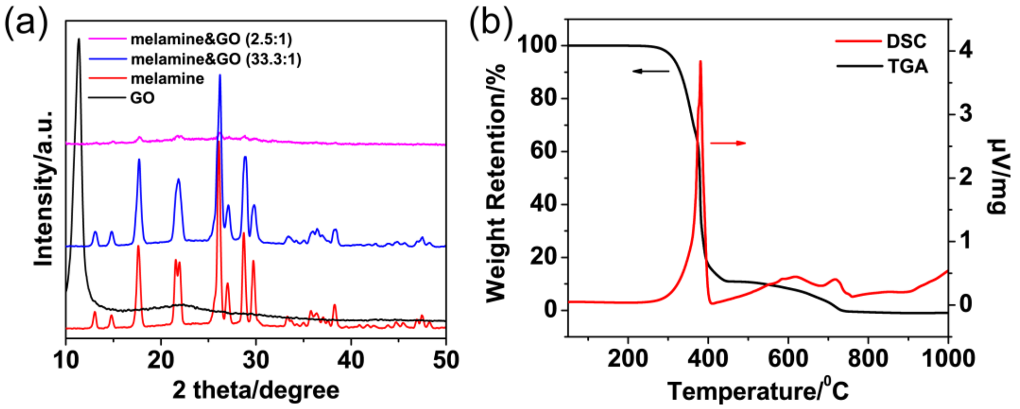

Figure S4. (a) XRD patterns of GO, melamine and their mixture with mass ratio of 2.5:1 and 33.3:1 (melamine to GO), respectively. Both GO and the mixtures were freeze dried from their suspensions. (b) TGA and DSC curves at a scan rate of 10 °C per minute in N_2 atmosphere, indicating melamine can be thermally decomposed into gas phase within a certain temperature range.

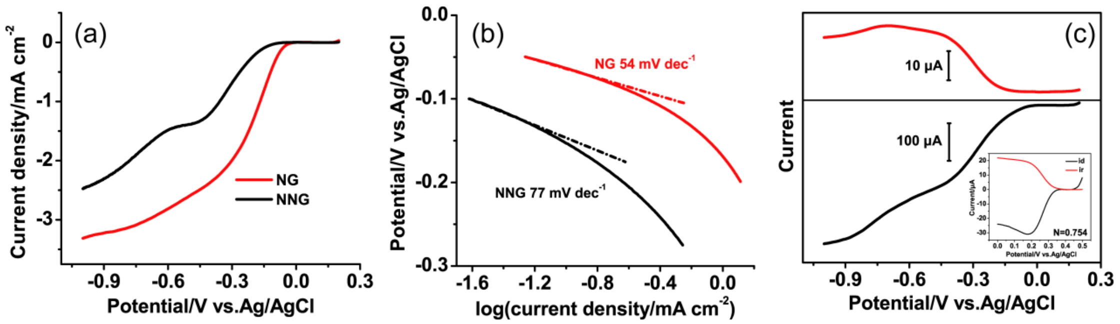

Figure S5. (a) RDE polarization curves of the NG and NNG (~loading 48 μg cm^{-2}) modified electrodes in O_2-saturated 0.1 M KOH at a rotation speed of 1000 rpm. (b) The tafel plots are derived from the results in Figure S5a. (c) RRDE polarization curves of NNG under the same testing conditions. Inset corresponds to the collection efficiency (N=0.754) of NNG modified GC electrode calculated by the ratio of ring current to disk current with 1 mM potassium ferricyanide as the probe in 0.1 M KCl.

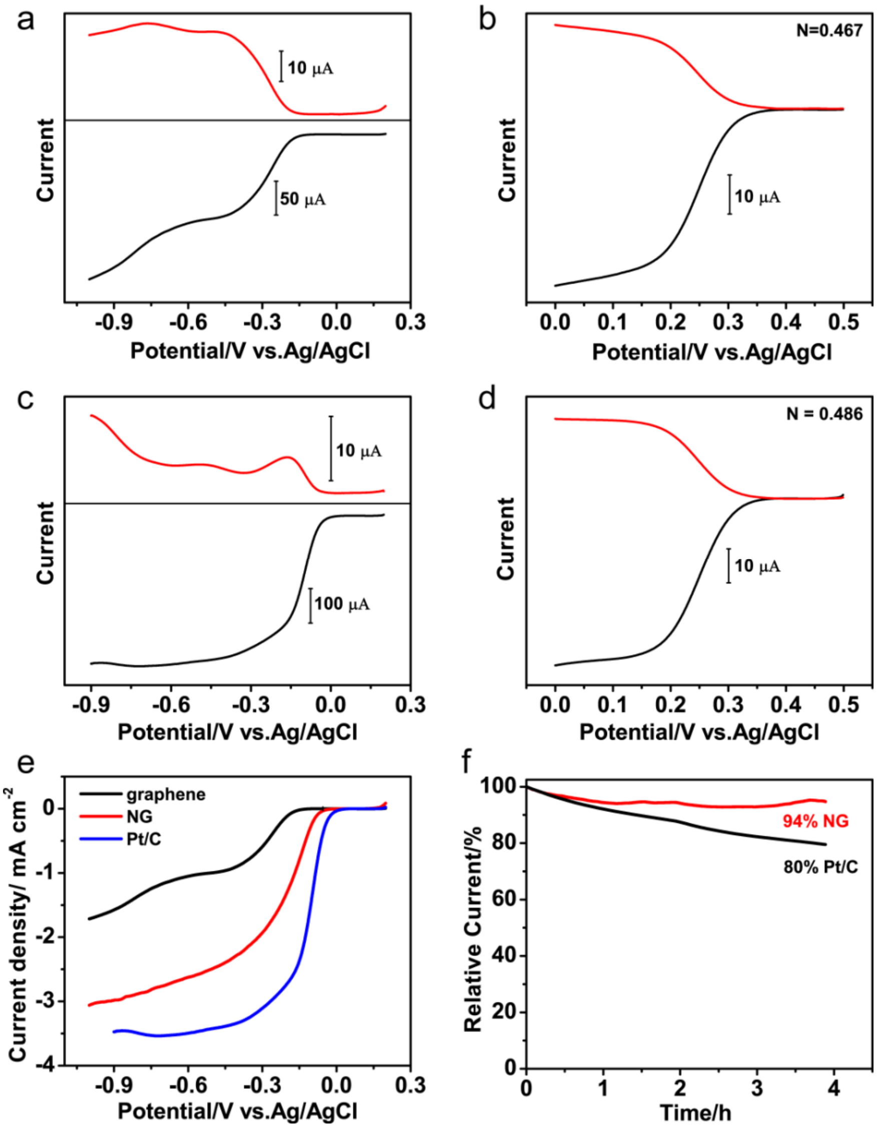

Figure S6. RRDE polarization curves of graphene (a) and Pt/C (40 wt% platinum on Vulcan XC-72) (c) in O_2-saturated 0.1 M KOH, 1000 rpm at a scan rate of 0.01 V/s with the measured collection efficiency of 0.467 (b) and 0.486 (d). On the basis of above experimental results, the calculated electron transfer number for graphene is 2.76 and for Pt/C is 3.99. (e) RDE polarization curves of graphene, NG and Pt/C (~loading 48 μg cm^{-2}). (f) The current-time chronoamperometric responses for ORR on NG (red curve) and Pt/C (black curve) at -0.4 V.

Table S1. The ORR performance of N-contained carbon catalysts in alkaline media.

Catalysts source	Method	Loading (μg cm^{-2})	Performance (V vs.Ag/AgCl)
In this study	CV<br>RRDE<br>Koutecky-Levich plots	~42.5	E_{onset}: -0.05, E_{peak}: -0.17<br>n: 3.66<br>n: 3.8-4
ACS Nano **2010**, 4, 1321	RDE<br>Koutecky-Levich plots	-	E_{onset}: ~ -0.18<br>n: 3.6-4
Angew. Chem. Int. Ed. **2010**, 49, 2565	CV<br>Koutecky-Levich plots	~25.5	E_{peak}: -0.26<br>n: 3.89
J. Am. Chem. Soc. **2011**, 133, 20116	CV<br>Koutecky-Levich plots	~84.4	E_{peak}: -0.25<br>n: 4
J. Am. Chem. Soc. **2011**, 133, 5182	CV<br>Koutecky-Levich plots	-	E_{onset}: -0.12, E_{peak}: -0.33<br>n: 3.72
Chem. Mater. **2011**, 23, 3987–3992	RDE<br>Koutecky-Levich plots	-	E_{onset}: ~ -0.13<br>n: 3.2-3.5
ACS Nano **2011**, 5, 4350.	CV<br>RRDE	-	E_{onset}: -0.1<br>n: 3.4-3.6
Adv. Funct. Mater. **2012**, 22, 3634	RDE<br>RRDE	-	E_{onset}: -0.04<br>n: 3.2-3.3
Adv. Mater. **2012**, 24, 5593	CV<br>RRDE	~102	E_{onset}: -0.13, E_{peak}: -0.22<br>n: 3.26-3.27
Angew. Chem. Int. Ed. **2012**, 51, 11371	RDE<br>Koutecky-Levich plots	~12	E_{onset}: -0.18<br>n: ~3.7
J. Am. Chem. Soc. **2012**, 134, 15	CV<br>Koutecky-Levich plots	~283	E_{onset}: -0.16, E_{peak}: -0.27<br>n: 3.6-4.4

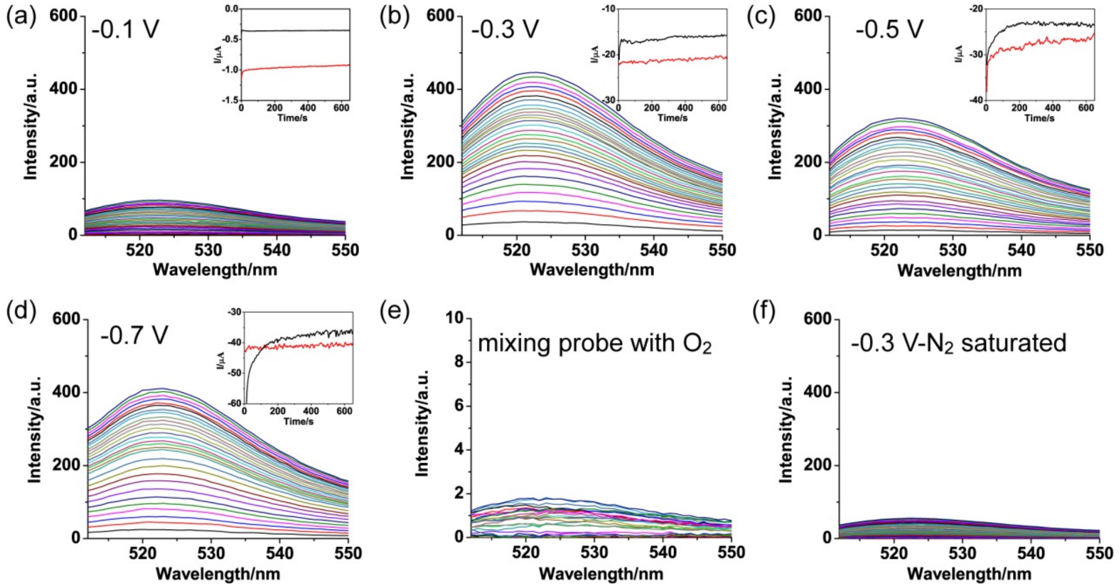

Figure S7. Fluorescence spectroelectrochemistry of 2.5 μM DCDHF in O_2-saturated 0.1 M KOH at -0.1 (a), -0.3 (b), -0.5 (c) to -0.7 V (d) and inset corresponds to the relatively current loss with/without adding DCDHF for 62% (a), 24% (b), 13% (c) and 8.8% (d). P.S.: if all DCDHF molecules are oxidized, the final fluorescent intensity would be far exceeding the measurement range under the same conditions. Controlled experiments suggest that the increased emission signals can be assigned to the oxygen intermediates originating from reduction process since without applying potential (e) or in N_2 atmosphere (with trace amount of O_2 residual, (f)), the increasing rate becomes much slower.

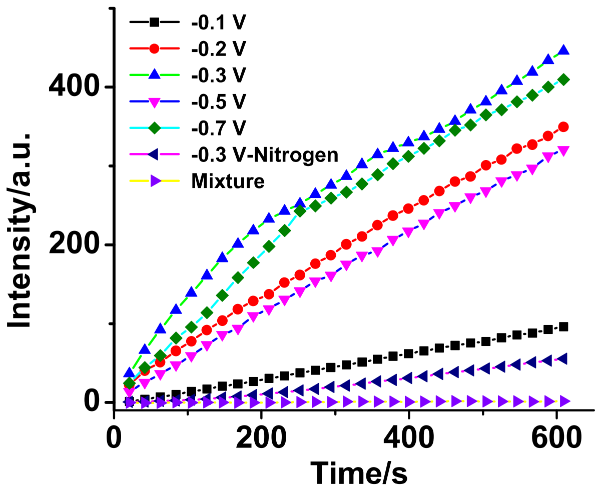

Figure S8. (c) The summarization of apparent increasing rates of fluorescent emission intensity.

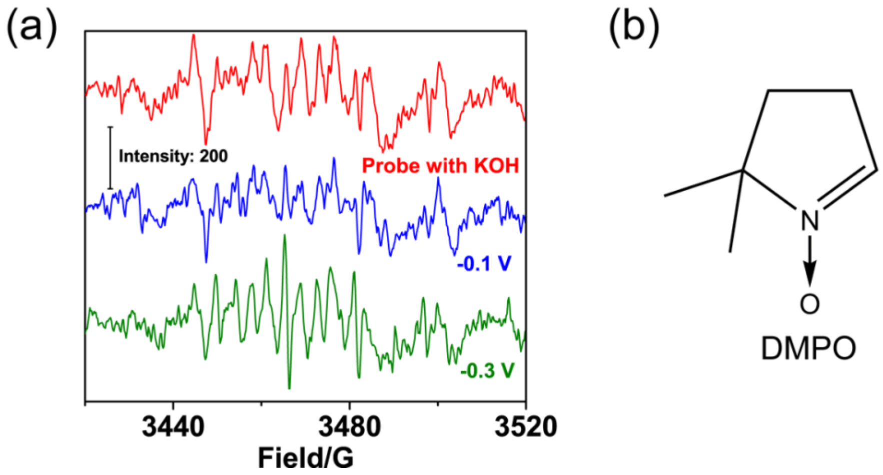

Figure S9. (a) EPR spectra of the oxygen intermediates captured during electrolyzing ORR. (b) Structure formula of the trapping probe, 5,5-Dimethyl-1-pyrroline N-oxide (DMPO).

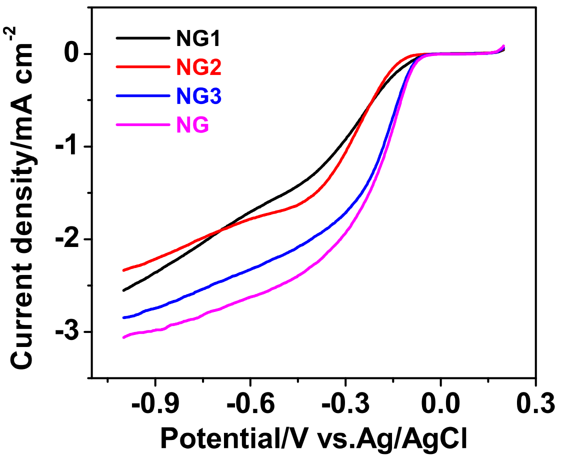

Figure S10. RDE polarization curves of NG1, NG2, NG3 and NG (~loading 48 μg cm^{-2}) at a scan rate of 0.01 V/s and a rotation speed of 1000 rpm in O_2-saturated 0.1 M KOH.

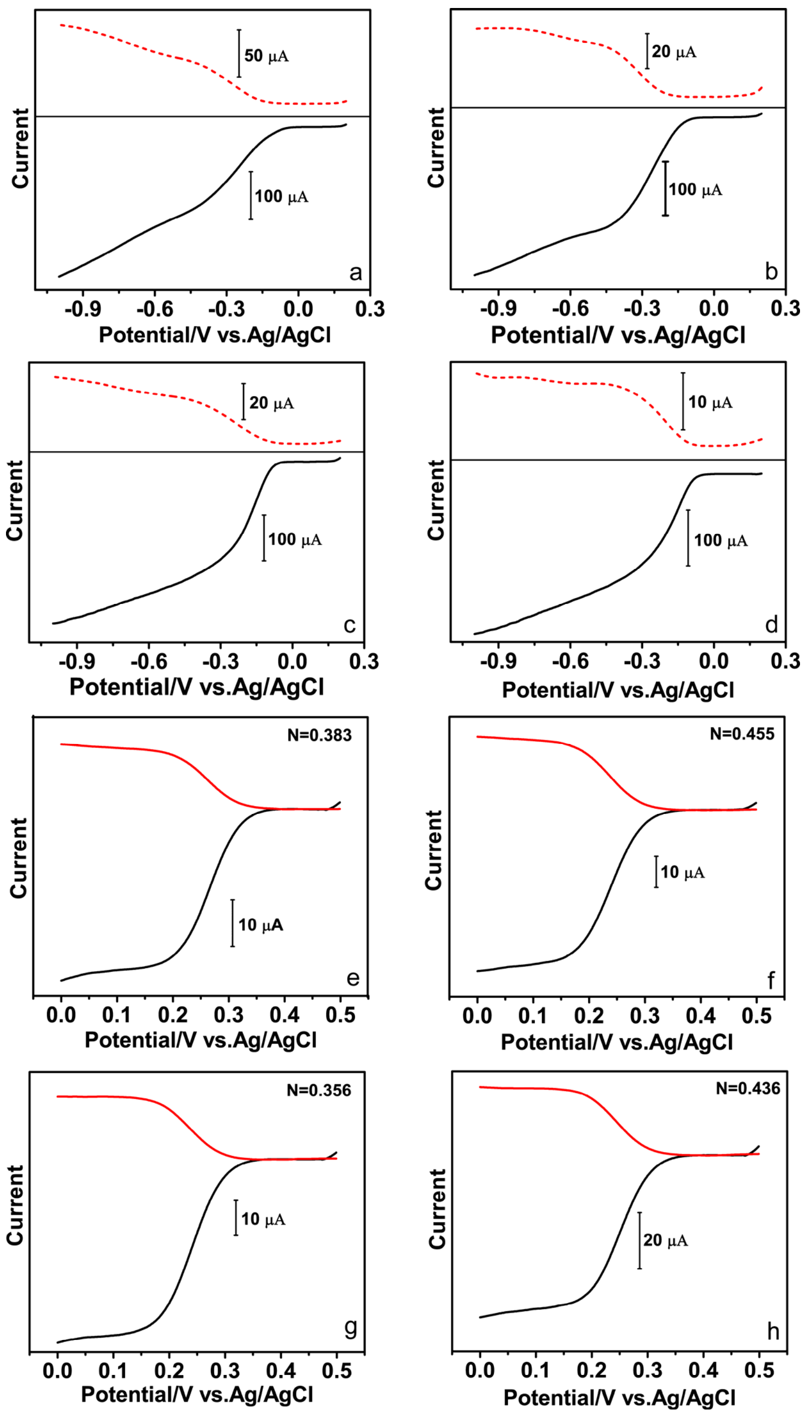

Figure S11. RRDE polarization curves of NG1 (a), NG2 (b), NG3 (c) and NG (d) at a scan rate of 0.01 V/s and a rotation speed of 1000 rpm in O_2-saturated 0.1 M KOH; Corresponding collection efficiency of NG1 (e), NG2 (f), NG3 (g) and NG (h) with 1 mM potassium ferricyanide as the probe in 0.1 M KCl.

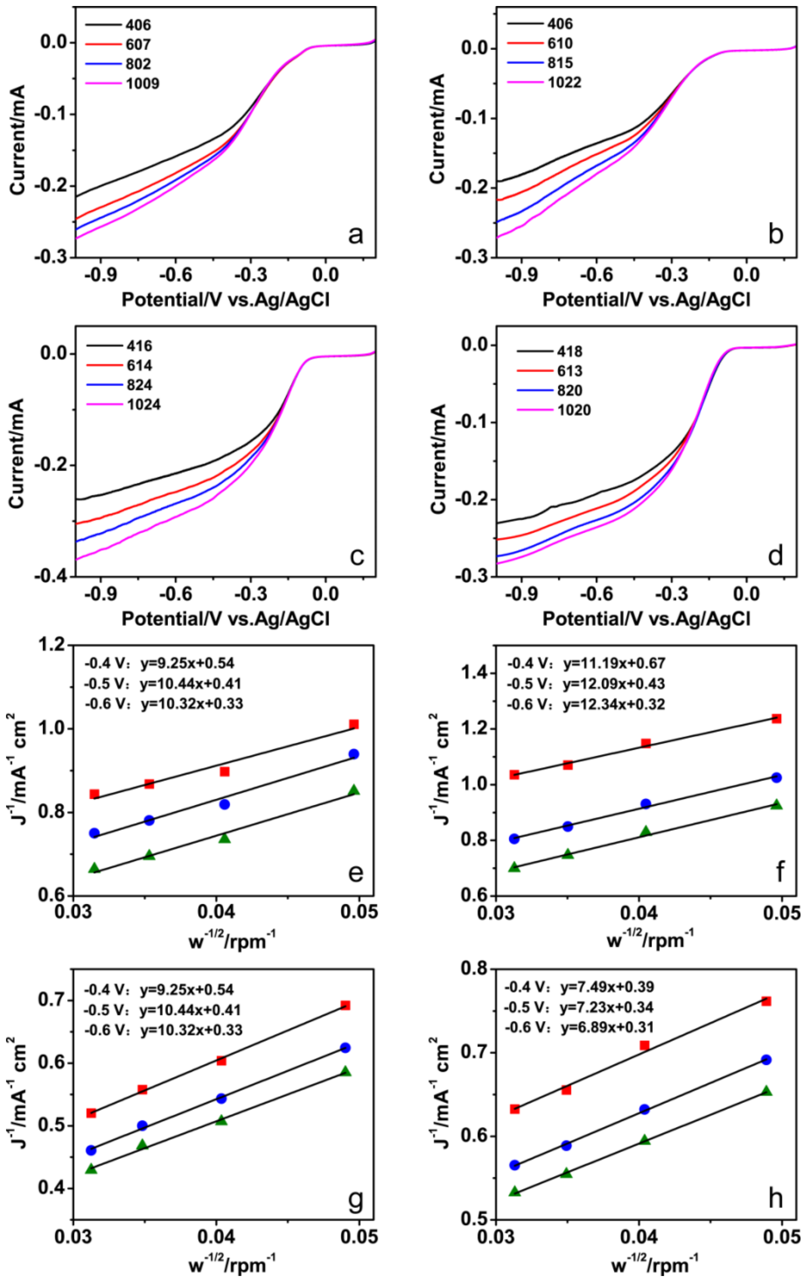

Figure S12. RDE polarization curves of NG1 (a), NG2 (b), NG3 (c) and NG (d) at a scan rate of 0.01 V/s in O_2-saturated 0.1 M KOH. By Koutecky–Levich plots with assuming $C_0 = 1.2 \times 10^{-6}$ mol cm^{-3}, $D_0 = 1.9 \times 10^{-5}$ cm s^{-1}, $\upsilon = 0.01$ cm^2 s^{-1}, the total electron transfer number is calculated to be 2.7 to 3 for NG1 (e), 2.3 to 2.5 for NG2 (f), 3 to 3.3 (g) for NG3 and 3.8 to 4 for NG (h).

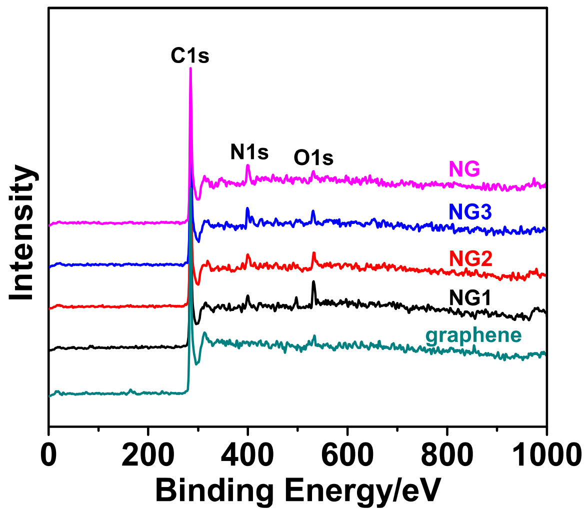

Figure S13. The XPS spectra of the graphene, NG1, NG2, NG3 and NG.

Table S2. Detailed atomic ratios of C, O and N elements contained in graphene, NG1, NG2, NG3 and NG.

	C at %	N at %	O at %
NG1	87.36	6.22	6.42
NG2	89.19	6.42	4.39
NG3	89.21	7.86	2.93
NG	88.35	8.54	3.11
graphene	97.46	-	2.54

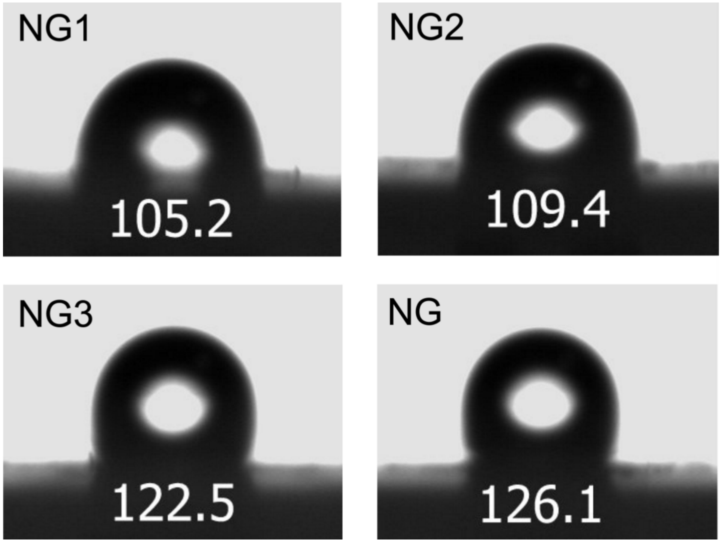

Figure S14. Contact angle measurements for NG1, NG2, NG3 and NG with water droplet as the probe. The contact angle for NG1, NG2, NG3 and NG are 105.2°, 109.4°, 122.5°, and 126.1°, respectively.

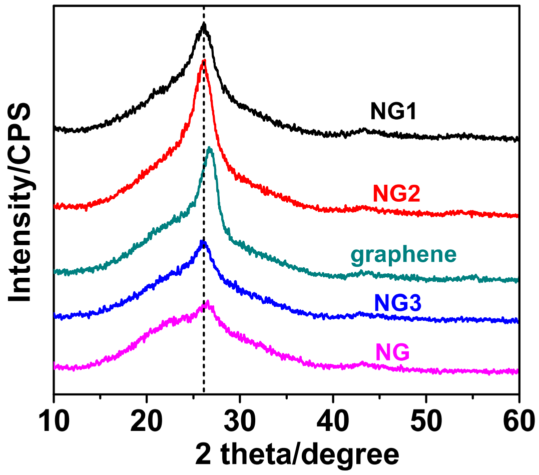

Figure S15. XRD patterns of NG1, NG2, NG3 and NG with pristine graphene as reference.

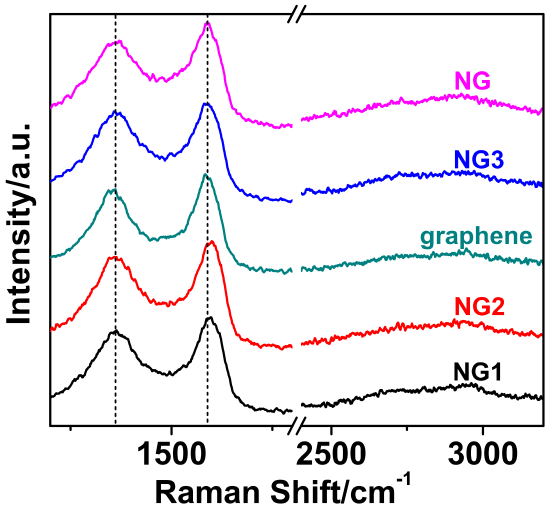

Figure S16. Raman spectra of NG1, NG2, NG3 and NG with pristine graphene as reference.

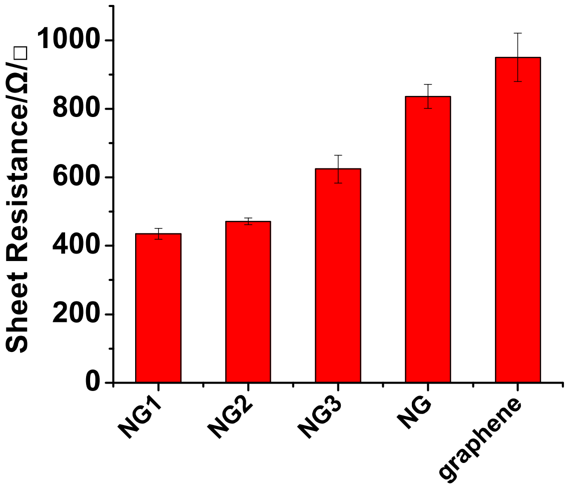

Figure S17. Sheet resistance of NG1, NG2, NG3 and NG with pristine graphene as reference.

Reference

(1) J. Wang, Y. Zhao, F. X. Ma, K. Wang, F. B. Wang, X. H. Xia, *J. Mater. Chem. B* **2013**, 1, 1406-1413.

(2) Z.-H. Sheng, L. Shao, J.-J. Chen, W.-J. Bao, F.-B. Wang and X.-H. Xia, *ACS Nano*, **2011**, 5, 4350-4358.

(3) L. R. F. Allen J. Bard, *Electrochemical Methods: Fundamentals and Applications* **2001**.

(4) Y. Liang, Y. Li, H. Wang, J. Zhou, J. Wang, T. Regier, H. Dai, *Nat. Mater.* **2011**, 10, 780-786.
